# Effect of Vitamin D Supplementation on Clinical Course and T Helper 17/ T-Regulatory Balance in Peripheral Blood of Patients with Crohn’s Disease

**DOI:** 10.5152/tjg.2023.22496

**Published:** 2023-05-01

**Authors:** Xiaodong Lin, Xiaoli Wu, Yini Zhang, Xinyi Shao, Hao Wu, Lingli Zhou

**Affiliations:** 1 Department of Pharmacy, The Second Affiliated Hospital and Yuying Children’s Hospital of Wenzhou Medical University, Wenzhou, China; 2 Department of Gastroenterology, The First Affiliated Hospital of Wenzhou Medical University, Wenzhou, China; 3 Department of Gastroenterology, The Second Affiliated Hospital and Yuying Children’s Hospital of Wenzhou Medical University, Wenzhou, China; 4 Department of Pathology, The Second Affiliated Hospital and Yuying Children’s Hospital of Wenzhou Medical University, Wenzhou, China

**Keywords:** Crohn’s disease, immune environment, inflammatory status, vitamin D

## Abstract

**Background:**

Vitamin D has anti-inflammatory properties and is involved in immune function, making it a potential therapy for Crohn’s disease. This study aimed to investigate the effects of vitamin D supplementation on immune function and the clinical efficacy of patients with Crohn’s disease.

**Methods:**

From September 2017 to September 2021, patients with Crohn’s disease were recruited and randomly divided into 2 groups: the routine treatment group (n = 52) and the vitamin D supplement group (n = 50). In addition to routine treatment, the vitamin D group received oral calcitriol capsule supplementation, while the routine treatment group did not receive any additional intervention. T helper 17/T-regulatory cell level, inflammatory indicators, and nutritional status were compared between the 2 groups, as well as mucosal healing under endoscopy and the life quality of patients.

**Results:**

C-reactive protein was significantly lower in the vitamin D treatment group compared to the routine treatment group (6.08 ± 2.72 vs. 18.91 ± 2.66, *P* < .05). Compared to the routine treatment group, the ratio of T helper 17/T-regulatory cells was significantly lower in the vitamin D group (0.26 ± 0.12 vs. 0.55 ± 0.11, *P* < .05). After vitamin D treatment, both of the average Crohn’s disease activity index score (from 319.7 ± 72.7 to 179.6 ± 48.5, *P* < .05) and simple endoscopic score for Crohn’s disease score (from 7.9 ± 2.3 to 3.9 ± 0.6, *P* < .05) were significantly decreased, while the Inflammatory Bowel Disease Questionnaire score was significantly increased (from 137.8 ± 21.2 to 158.1 ± 25.1, *P* < .05).

**Conclusions:**

Vitamin D has the potential to improve the inflammatory status and immune environment of patients with Crohn’s disease, which can reduce the level of inflammatory factors and help the recovery of symptoms, thus improving the clinical course and quality of life in Crohn’s disease patients.

**Main Points**

- Vitamin D has the potential to improve the inflammatory status of patients with Crohn’s disease (CD).
- Vitamin D has the potential to improve the immune environment of patients with CD.
- Vitamin D can improve the clinical efficacy and life quality of CD patients.

## INTRODUCTION

Crohn’s disease (CD) is a chronic, nonspecific, and recurrent inflammatory bowel disease associated with immune disorders.^1^ Patients with CD frequently experience gastrointestinal complications like bowel strictures, fistulae, anemia, and malnutrition, as well as extraintestinal complications such as pyoderma gangrenosum, uveitis, and venous thrombosis.^2^ Crohn’s disease is a chronic disease with a high recurrence rate, and it is difficult to cure, which significantly impacts the patient’s life expectancy and quality of life. T helper 17 (Th17) and T-regulatory (Treg) cells are 2 vital immune cells that typically maintain the balance of the body.^3^ However, dysregulation of Th17/Treg balance can contribute to the development of autoimmune and inflammatory diseases, including CD. Research has shown that an imbalance in the Th17/Treg ratio is involved in the pathogenesis and progression of CD.^4^

The main therapies used to treat CD contain medication, nutrition, and surgical treatment. Steroids, glucocorticoids, aminosalicylic acid, and immunosuppressants are commonly used in clinical treatment.^5^ The objective of treatment is to induce and sustain remission, prevent relapse, and mitigate long-term complications, thus enhancing the quality of life of patients.^2^ However, current treatment strategies have limitations. It was reported that prolonged steroid use is associated with numerous adverse effects and increased mortality.^6^ Therefore, optimizing clinical management concerning treatment efficacy and side-effect profiles is crucial. Complementary and alternative therapies may serve as adjunctive measures to ameliorate these limitations.^7^

Recent research suggested that nutrition-related treatments offer significant benefits as a cost-effective and non-specific alternative to immune system suppression in CD.^8^ Vitamin D (VitD) plays a crucial role in immune regulatory functions, and deficiency of VitD can lead to osteochondrosis, osteoporosis, high blood pressure, and heart disease.^9^ Olliver et al^10^ demonstrated that VitD modulates immune function by interacting with innate and adaptive immunity, regulating inflammation, and enhancing intestinal barrier integrity. Individuals with low VitD levels have a higher likelihood of requiring steroids, biological products, emergency department visits, hospitalization, and surgery.^11^

Our team has already analyzed the relationship between serum 25(OH)D level and CD. We found that the average level of 25(OH)D in the CD group was significantly reduced, and 25(OH)D deficiency may be an independent risk factor for CD susceptibility. What is more, a reduced level of 25(OH)D increases the individual’s risk of developing CD.^12^ Therefore, in this present study, we were interested in exploring whether VitD supplementation can improve the condition in CD patients through a prospective, open, and controlled clinical study.

## MATERIALS AND METHODS

### Study Subjects

From September 2017 to September 2021, patients with CD recruited from the Gastroenterology Department were selected as subjects for this study. All patients submitted written informed consents to the collection and use of clinical samples, and the study was approved by the Scientific Ethics Committee of the First Affiliated Hospital of Wenzhou Medical University, and the Scientific Ethics Committee ofthe Second Affiliated Hospital and Yuying Children’s Hospital of Wenzhou Medical University. The side effects of the drugs used in this study have been notified.

The inclusion criteria are as follows: (1) patients should be more than 18 years old and (2) the diagnostic criteria should be in line with the standard of 2018 “consensus on the diagnosis and treatment of inflammatory bowel disease in China.”^13^

The exclusion criteria are as follows: (1) patients with incomplete clinical and follow-up data; (2) patients with obvious infection (including pulmonary tuberculosis, viral hepatitis, or other potential infection), organic heart damage, diabetes, and elevated glutamic pyruvic transaminase (GPT).

### Intervention and Control

The trial was designed as a prospective, randomized, and controlled trial in a parallel experiment. One hundred two patients with CD were randomly divided into the routine treatment (RT) group (n = 52) and the VitD supplement group (n = 50), using a random number table method. Twenty-six patients in the RT group were treated with methylprednisolone (0.75-1 mg/kg/day) and azathioprine. For patients whose symptoms were in remission, the dose of methylprednisolone was decreased by 5 mg every week until 20 mg per day, and then the amount was decreased by 2.5 mg every week. Azathioprine (1.5-2.5 mg/kg/day) was fully given for 30 weeks. The remaining 26 patients received intravenous infliximab (IFX, 5 mg/kg) at 0, 2, 6, and 14 weeks, as induced remission, followed by consolidation therapy twice every 8 weeks (the last time was at 30 weeks). The VitD supplement group received oral administration of calcitriol capsules in addition to conventional treatment. The dosage was 1 calcitriol soft capsule (rocaltrol, 0.25 μg/capsule) in the morning and 1 in the evening for a total of 0.5 μg a day for 30 weeks, and the other conditions were the same. Among the 50 patients, half of them were treated with methylprednisolone and azathioprine. The other 25 patients received IFX intravenously. All the patients were strictly screened before IFX treatment, including dermal purified protein derivative of tuberculin test (PPD test), T cell spot test, chest x-ray, or chest computed tomography scan to rule out tuberculosis. Patients were educated on lifestyle-related habits, including strict abstinence from smoking and alcohol, proper daily eating and rest habits, and exposure to sunlight.

### Sample Collection

At the time of recruitment (week 0) and at the end of treatment (week 30), patients underwent colonoscopy examination, and intestinal biopsies were taken from the intestinal region. Biopsies were collected and stored in formaldehyde or liquid nitrogen for further analysis. Before and after the trial, the whole blood of the patients was collected, and the immune indicators were detected.

### Laboratory Parameters

The following indexes were detected before and 30 weeks after treatment: blood routine, aspartate aminotransferase (AST), GPT, body mass index (BMI), albumin (ALB), erythrocyte sedimentation rate (ESR), VitD, parathormone (PTH), calcium, serum phosphorus, and C-reactive protein (CRP).

### T Helper 17 and T-Regulatory Cell Detection

#### T Helper 17 Cell Assay:

Two test tubes were prepared, labeled as homotype tube and positive tube respectively, and to each tube, 20 μL of CD3 labelled with fluorescein isothiocyanate (CD3-FITC) and CD8 labelled with phycoerythrin conjugated cyanine 7 (CD8-PE-Cy7) antibodies was added. Then, 50 μL of the whole blood which had been stimulated and cultured for 5 hours was mixed and incubated at room temperature for 15 minutes. To this, 800 μL of the hemolysin was added and was fully mixed in the dark for 3 minutes. The mixture was transferred to a 37°C water bath and incubated for 5 minutes, and then 1 mL of phosphate-buffered saline (PBS) was added, mixed, and centrifuged at 1000 rpm for 5 minutes. The supernatant was discarded and the procedure was repeated once. After adding 1 mL membrane breaker to mix, break the membrane for 45 minutes, centrifuge at 1000 rpm for 5 minutes, then discard the supernatant and add the antibody labeled homotype control (–) and Th17 (+), to mix it well and incubate at room temperature, away from light for 15 minutes. One milliliter of PBS buffer was added, fully mixed, and centrifuged for 5 minutes, and then the supernatant was discarded. The procedure was repeated once and again the supernatant was discarded, and an appropriate amount of PBS was added and mixed well and tested on the machine. The results of Th17 cells were evaluated as the percentage of CD3^+^/CD8^–^/IL17^+^ cells.

#### T-Regulatory Cell Assay:

Two test tubes were prepared. Twenty microliters of immunoglobin G1 labelled with allophycocyanin (IgG1-APC) and IgG2a-Alexflout672 antibodies was added to the first tube as homotype control, 20 μL of TregCocktail (CD4-FITC, CD25-APC, and CD127-Alexflour672) was added to the second tube, then ethylenediamine tetraacetic acid dipotassium salt (EDTA-K2) anticoagulant peripheral blood was added. The solution was mixed fully with a whirlpool mixer, incubated at room temperature, and light was avoided for 15 minutes. To this, 800 μL of the hemolysin was added and was fully mixed in the dark for 3 minutes. The mixture was transferred to a 37°C water bath and incubated for 5 minutes, and then 1 mL of PBS was added, mixed, and centrifuged at 1000 rpm for 5 minutes. The supernatant was discarded and the procedure was repeated once. The supernatant was discarded and an appropriate amount of PBS was added and mixed well and tested on the machine. The results of Treg cells were evaluated as the percentage of CD4^+^/CD127^low^/CD25^+^ cells.

### Clinical Efficacy

Crohn’s disease activity index (CDAI) was used to evaluate the severity of disease activity and the curative effect: (i) Active CD was defined as a CDAI score of ≥150; (ii) Clinical remission was defined as a CDAI score of <150; (iii) Clinical efficacy was defined as a decrease in CDAI score of >70 points compared to the control group, or a decrease of >25% of the total CDAI score of the control group; and (iv) Clinical inefficacy was defined as an increase in CDAI score of >70 points compared to pre-treatment levels with a total CDAI score of >175, or an increase of >35% compared to the CDAI score of the control group, or the use of additional medication to control symptoms. Effective treatment was defined as clinical remission or clinical efficacy.

### Endoscopy and Histopathological Examination

Endoscopy (including colonoscopy and balloon enteroscopy) was performed before and after treatment (30 weeks), and the simplified endoscopic score^14^ was adopted to evaluate the efficiency of VitD. The ulcer-related scores in the simple endoscopic score for Crohn’s disease (SES-CD) were defined as follows: ulcer size (no ulcer—0; ulcer diameter 0.1-0.5 cm—1; ulcer diameter >0.5-2.0 cm—2; ulcer diameter >2.0 cm—3), and the proportion of intestinal ulcer (no intestinal ulcer 0; <10% is considered 1; 10%-30% is considered 2; >30% is considered 3). Two senior endoscopic physicians in the Department of Gastroenterology in our hospital scored independently. If there is any objection, the third senior endoscopic physician participates in the discussion and draws a conclusion. For pathological examination, the intestinal biopsies were fixed overnight with 4% paraformaldehyde and then embedded in paraffin. After dewaxing and rehydration, paraffin sections (5 μm) were stained with hematoxylin–eosin (HE) for routine histopathological observation.

### Assessment of Life Quality

The Inflammatory Bowel Disease Questionnaire (IBDQ) was used to evaluate the life quality of all subjects.^15^ The scope of the questionnaire includes 4 areas: symptoms related to bowel disease (such as abdominal pain and diarrhea), general condition (such as fatigue and sleep disorders), social function (such as participation in work or social interaction), and emotional status (depression and exasperation). Each field contains 8 questions with a total of 32 questions. Each question has answers in varying degrees from 1 to 7. One represents the most negative impact on the quality of life and 7 represents the least negative impact on the quality of life. The total score is 224, and the higher the score, the higher the quality of life.

### Statistical Analysis

All calculations were performed using Statistical Package for Social Sciences (SPSS) version 17.0 software for Windows (SPSS, Inc., Chicago, IL, USA). Quantitative data with normal distribution and homogeneous variances are presented as mean ± SD. Comparisons between the 2 groups were performed using an independent sample *t*-test; categorical data were analyzed using the chi-square test and presented as the rate. A *P*-value <.05 was considered indicative of a statistically significant difference.

### Adverse Reactions

Any adverse events during treatment (such as digestive tract symptoms, infusion reactions, and liver function) were recorded.

## RESULTS

### Baseline Characteristics of the Study Group

A total of 102 CD patients were enrolled in this study. The VitD group consisted of 50 patients, of which 28 were male with an average age of 29.8 ± 10.7 years. The average CDAI score was 319.7 ± 72.7. The RT group comprised 52 patients, of which 27 were male with an average age of 28.1 ± 9.8 years and the average CDAI score in RT group was 311.9 ± 68.4. The clinical characteristics of the patients are shown in Table 1, with no significant differences between the 2 groups (*P* > .05).

### Effects of Vitamin D on Biochemical and Inflammatory Parameters

**Figure 1.**
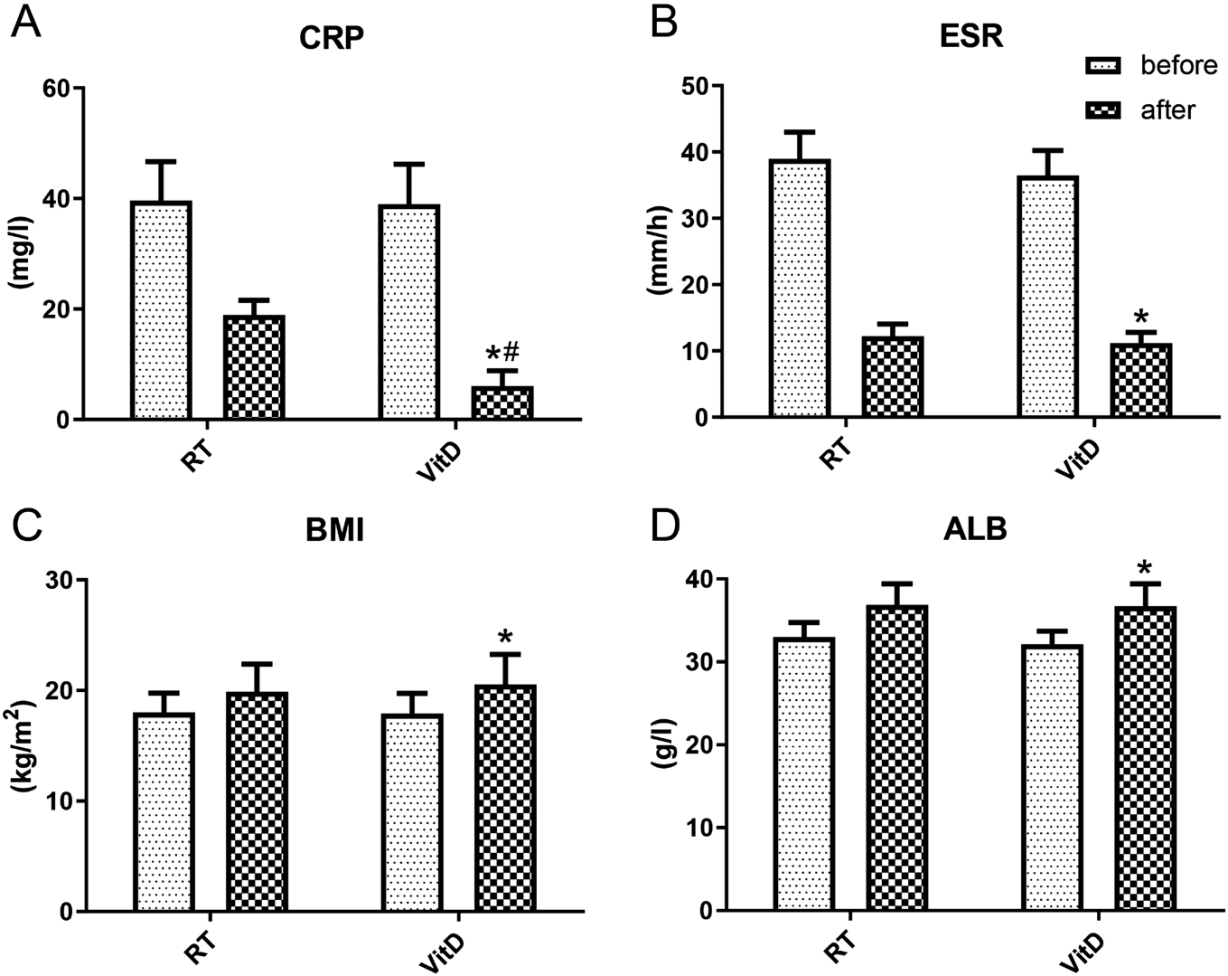
Laboratory parameters and body mass index (BMI) of patients treated with vitamin D. (A) C-reactive protein (CRP). (B) Erythrocyte sedimentation rate (ESR). (C) BMI. (D) Albumin (ALB). *P < .05 versus the vitamin D (VitD) treatment group (before treatment), ^#^*P* < .05 versus the RT group.

**Supplementary Figure 1.Level of vitamin D (VitD), parathormone (PTH),:**
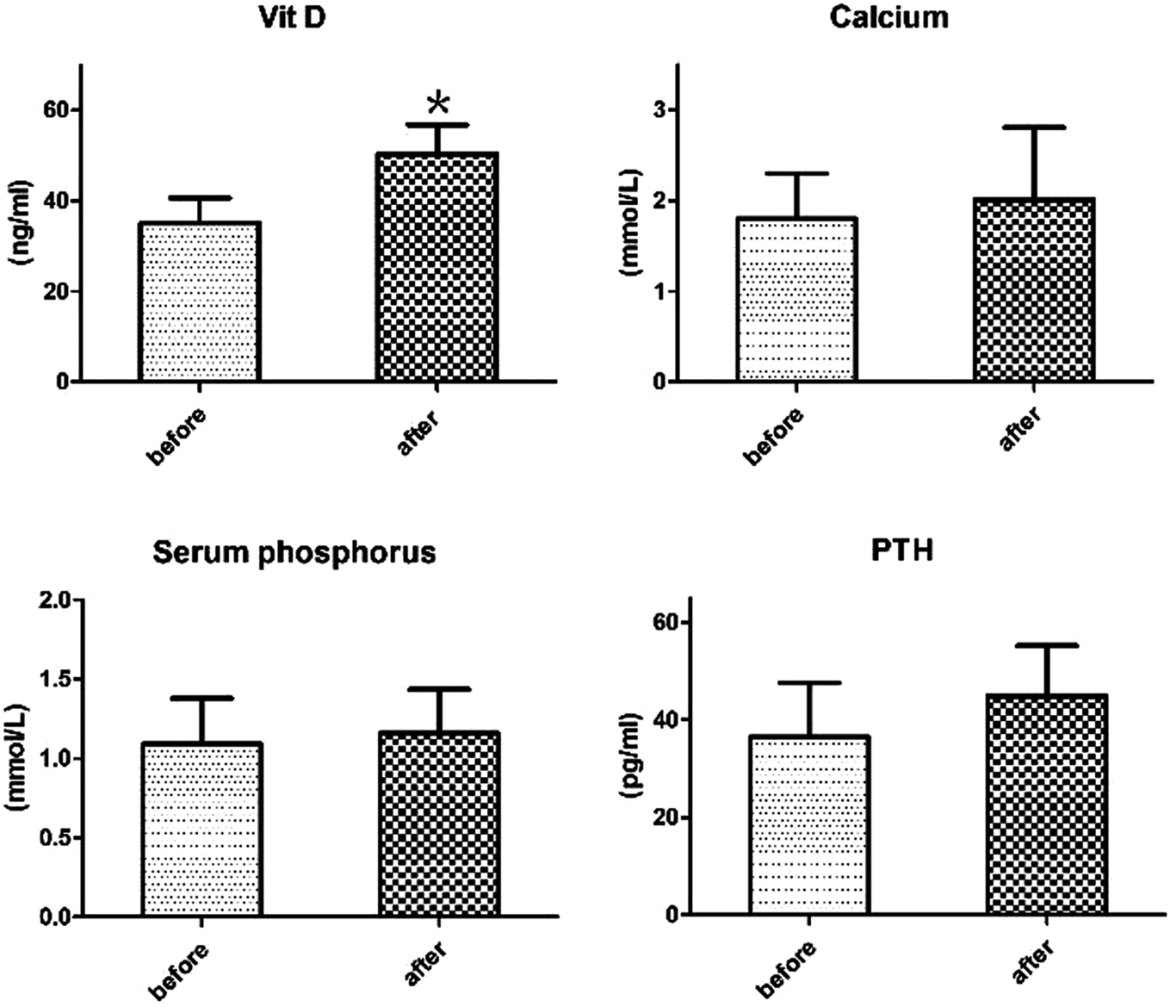
calcium and serum phosphorus of patients treated with VitD. *P < .05 versus the VitD treatment group (before treatment).

As shown in Figure 1 and Supplementary Table 1, after 30 weeks of treatment in the VitD group, the levels of CRP (38.99 ± 7.23 vs. 6.08 ± 2.72) and ESR (36.43 ± 3.78 vs. 11.18 ± 1.62) decreased significantly (*P* < .05), while the levels of BMI (17.92 ± 1.81 vs. 20.53 ± 2.72) and ALB (32.11 ± 1.62 vs. 36.72 ± 2.69) increased significantly (*P* < .05). In particular, it is worth noticing that the level of CRP in the VitD group decreased more significantly than that in the RT group (6.08 ± 2.72 vs. 18.91 ± 2.66, *P* < .05). However, no significant change was detected in AST, GPT, and other laboratory parameters between the 2 groups. Vitamin D level was measured in patients before and after administration, and we found that the normal clinical limit (>75 ng/mL) was not reached after treatment, although there was a statistical difference (Supplementary Figure 1).

### Effects of Vitamin D on Immunological Parameters

**Figure 2.**
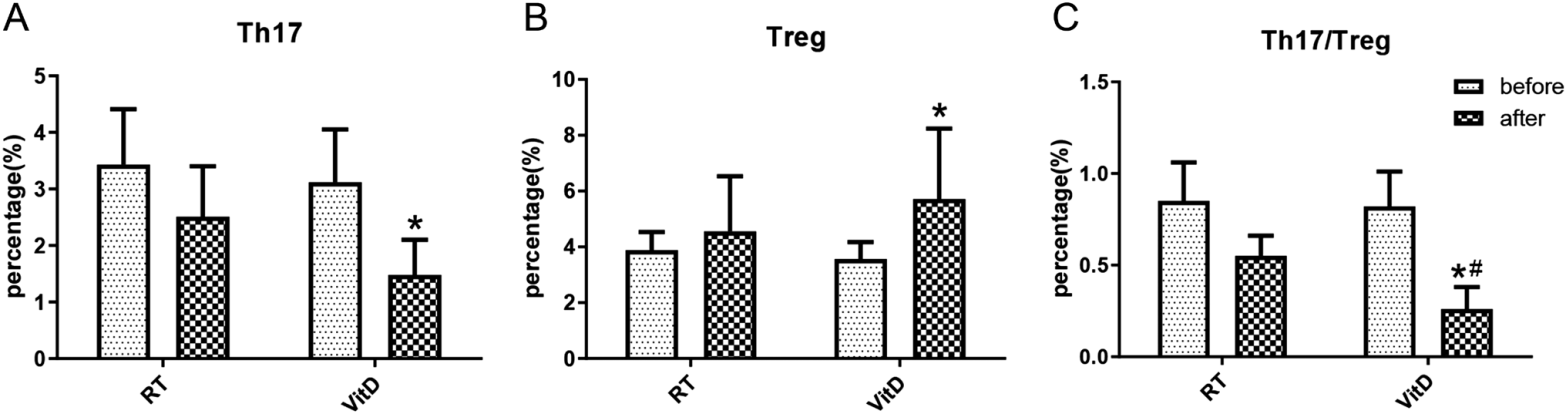
The proportion of T helper (Th17) cells and T-regulatory (Treg) cells in patients treated with vitamin D. (A) The percentage of Th17 cells. (B) The percentage of Treg cells. (C) The ratio of Th17/Treg cells. *P < .05 versus the VitD treatment group (before treatment); ^#^*P* < .05 versus the RT group.

In order to explore the effect of VitD on immune function, we detected the proportion of Th17 and Treg cell level. The results showed that after 30 weeks of VitD supplementation, the proportion of Th17 cells decreased significantly (3.12 ± 0.93 vs. 1.48 ± 0.62, *P* < .05), while the proportion of Treg cells increased significantly (3.56 ± 0.61 vs. 5.71 ± 2.53, *P* < .05). In addition, as seen in Figure 2 and Supplementary Table 2, the ratio of Th17/Treg decreased significantly in both different treatment groups, and the VitD group was better than the RT group in reducing the Th17/Treg level (0.26 ± 0.12 vs. 0.55 ± 0.11, *P* < .05). The results suggested that VitD can regulate the balance of Treg/Th17 cells.

### Effects of Vitamin D on Histopathology

**Figure 3.**
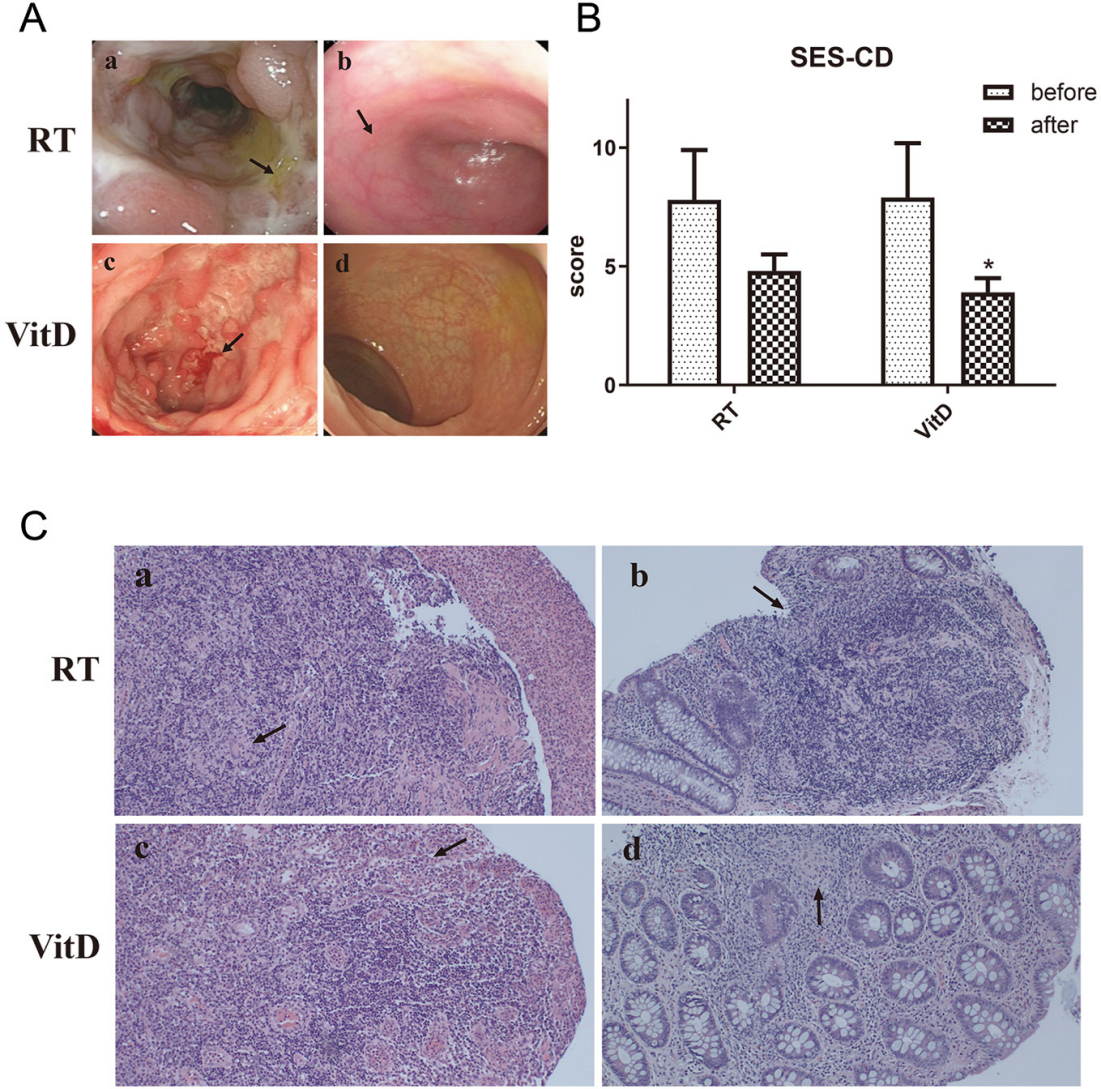
Changes in the pathology of colonic tissue in patients treated with vitamin D (VitD). (A) Representative endoscopic images of the routine treatment (RT) group and VitD group (before and after treatment). (B) Simplified endoscopic scores of the RT group and VitD group (before and after treatment, *P < .05 versus the VitD treatment group (before treatment). (C) Hematoxylin–eosin staining of colon tissues (×100) of the RT group and VitD group (before and after treatment).

To investigate the impact of VitD on CD patients directly, we compared the pathological changes resulting from 2 different treatments. Colonoscopic images (Figure 3A) showed the presence of multiple large, irregular longitudinal ulcers before treatment, while no significant ulcers were observed after VitD treatment. Notably, some aphthous ulcers were occasionally observed after RT treatment. Furthermore, the SES-CD score decreased significantly after VitD supplementation (7.9 ± 2.3 vs. 3.9 ± 0.6, *P* < .05, Figure 3B, Supplementary Table 3). Consistently, the HE staining results (Figure 3C) showed that inflammatory exudation and granulation tissue hyperplasia as well as non-caseous granulomata before treatment, and mucosa in the VitD group was almost completely healed, while mucosal aphthous erosion still can be observed under RT treatment.

### Effects of Vitamin D on Clinical Course and Life Quality

**Figure 4.**
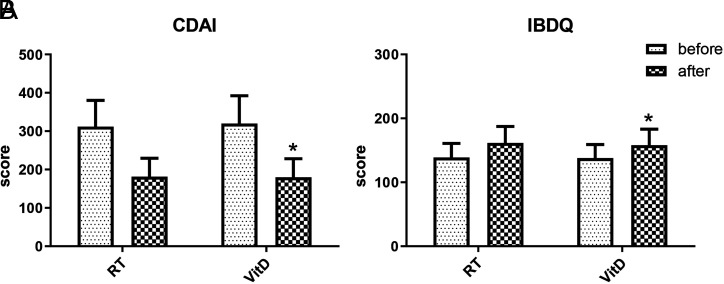
Crohn’s disease activity index (CDAI) and Inflammatory Bowel Disease Questionnaire (IBDQ) score of patients treated with vitamin D (VitD) supplement. (A) CDAI. (B) IBDQ. *P < .05 versus the vitamin D (VitD) treatment group (before treatment).

To investigate whether VitD can improve clinical efficacy and quality of life in CD patients, CDAI and IBDQ were evaluated between the 2 different treatments. As shown in Figure 4 and Supplementary Table 3, we found that CDAI score decreased significantly after VitD treatment (319.7 ± 72.7 vs. 179.6 ± 48.5, *P* < .05), while IBDQ score increased significantly (137.8 ± 21.2 vs. 158.1 ± 25.1, *P* < .05). However, compared with the RT group, the VitD group showed no advantage in CDAI and IBDQ score after treatment (*P* > .05).

### Adverse Drug Reactions

Adverse effects of VitD supplementation were determined by testing levels of PTH, calcium, and serum phosphorus levels, and no significant differences were found (Supplementary Figure 1). Among the patients in the VitD group, 3 patients had a decrease in white blood cell (WBC) count while taking azathioprine (AZA)(WBC count <4 × 10^9^/L), but when the dosage of AZA was reduced, we found that the level of WBC returned to normal. In addition, 3 patients had infusion reactions during IFX injection, such as dizziness, chest tightness, cold sweat, and transient rash all over the body; symptoms disappeared after drug withdrawal and no adverse reactions occurred after reinjection. Similarly, in the RT group, 5 patients had a decrease in WBC while taking AZA (WBC count <4 × 10^9^/L), but when the dosage of AZA was reduced, the level of WBC returned to normal. Two patients had infusion reactions while taking IFX injection; symptoms disappeared after drug withdrawal and there was no adverse reaction when used again.

## DISCUSSION

Currently, intestinal mucosal immune reactions and dysfunction are considered the primary factors leading to the pathogenesis of CD. Treatment options for CD include hormones combined with immunosuppressants and administration of IFX, a tumor necrosis factor-α (TNF-α) inhibitor that is the first biologic agent formally applied for CD treatment. The clinical use of TNF-α inhibitors has been shown to be associated with higher levels of VitD in patients with CD.^16^ Despite the predominance of immune suppression therapies, recent attention has been focused on the importance of nutrition therapy in treating and preventing diseases. Nutritional interventions offer a cost-effective alternative to immune suppression by restoring immune system balance. Previous data from our research group revealed that patients with CD were lack of VitD, and we thus sought to investigate whether VitD supplementation could improve their clinical condition. In this study, we demonstrated that VitD supplementation significantly lowers inflammatory indices and ameliorated inflammatory responses in CD patients. Meanwhile, the ratio of Th17/Treg significantly decreased in both treatment groups, while the VitD group showed superior capacity for lowering the Th17/Treg level compared to the RT group. This finding indicated that VitD supplementation can restore the balance of Th17 and Treg cells in CD patients, leading to improved autoimmune regulation and alleviated intestinal inflammation. Colonoscopy results revealed that VitD treatment was significantly more effective in reducing ulcers and repairing intestinal mucosa compared to the control group. Moreover, we found that VitD treatment improved the clinical efficacy and life quality of CD patients, as evidenced by CDAI and IBDQ scores.

At present, the pathogenesis of CD remains unclear, but evidence suggested that it is caused by abnormal T cell immune responses, which are triggered by inflammatory response of intestinal microorganisms in individuals with susceptible genes.^17^ Some studies have suggested that CD is initiated by autoimmune-related Th17 and Th1 cells, and the dysregulation of Th17/Treg response is a fundamental aspect of its pathogenesis.^18,19^ Previous studies have focused on the balance of Th1/Th2 cells, but the Th17/Treg cell balance can better reflect the body’s immune response to autoantigens or foreign antigens, explaining the pathogenesis and pathophysiological changes of autoimmune diseases and chronic inflammatory diseases. The Treg/Th17 imbalance results in abnormal innate immune response, culminating in immune activation and the production of proinflammatory cytokines, and finally magnifies and chronicles the inflammation. Chao et al^20^ observed a significant decrease in the ratio of Treg cells and mRNA expression in the peripheral blood of patients with CD, along with a significant increase in the ratio of Th17/Treg. During the 2-year follow-up, it was demonstrated that the higher Th17/Treg ratio had a higher tendency of recurrence. Our study showed that low-dose VitD can elevate the number of Treg cells, greatly declining the ratio of Th17/Treg and multiple ulcers in the intestinal mucosa, identifying VitD as a potential inexpensive immunomodulator that can provide a new target for the treatment of CD. Interestingly, Schäffler et al^21^ reported that VitD may positively affect CD by regulating the composition of intestinal bacteria and increasing the abundance of potentially beneficial strains. Additionally, recent research demonstrated that VitD was associated with disease activity and intestinal inflammation, which may be involved in the Treg/Th17 balance and the expression of gut tight junction (TJ) proteins.^22^ Accordingly, we speculated that VitD may improve the T cell immune response by affecting the intestinal microorganisms, and this hypothesis needs to be verified in further research.

Malnutrition is highly prevalent in CD, with 86.4% of CD surgical inpatients exhibiting malnutrition.^23^ The interaction between nutrients and inflammatory factors can further promote disease development.^24^ Our research confirmed that the average BMI levels in the VitD group were significantly higher after treatment, indicating a possible relationship between improved nutritional status and immune function recovery. Furthermore, serum CRP is associated with rheumatoid arthritis, obesity, diabetes, cardiovascular disease, and other diseases^25-27^ and can better reflect the inflammatory activity. Previous studies have shown that patients with a normal CRP level easily achieved a significant clinical response and had a better prognosis than patients with CRP >5 mg/L.^28,29^ Our results also indicated that, after treatment for 30 weeks, the level of CRP decreased sharply after VitD supplementation. The difference between the 2 groups suggested that VitD greatly enhanced the anti-inflammatory effect of drugs. Under different treatment methods, the levels of ESR and ALB recovered significantly in the 2 groups, but there was no significant difference between the 2 therapies.

Serological indicators such as ESR and CRP can reflect the degree of inflammation to some extent, and the clinical use of CDAI combined with CRP, ESR, and other inflammatory indicators comprehensively assesses disease activity in CD patients. Crohn’s disease activity index score is one of the commonly used indexes to evaluate the disease activity of patients with CD.^30^ In this experiment, we observed that the CDAI score significantly decreased after treatment in the VitD group, which suggested that the condition of CD has improved. In addition, we also made a questionnaire on the life quality of inflammatory bowel disease. As a result, our study demonstrated that the IBDQ score was significantly elevated after VitD treatment. It is known that the SES-CD is a simplified tool for measuring the severity of endoscopic disease. In clinical trials of CD, endoscopic indicators are increasingly adopted to evaluate the efficacy of various therapeutic drugs in inducing and maintaining mucosal healing, which are considered to be a gold standard for indicating the existence of active enteritis. Besides, we can see the SES-CD scores in the VitD treatment group were significantly lower than that before treatment (*P* < .05). Interestingly, Schoepfer et al reported that the SES-CD score was positively correlated with CRP, blood leukocytes, and the CDAI.^31^

The present study aimed to evaluate the potential benefits of adding VitD to conventional treatment for CD, and we found that the additional use of VitD can be beneficial for patients, but its specific mechanism remains to be further investigated. In the future, we plan to use molecular biology techniques to explore the specific mechanisms by which VitD exerts its positive effects in conventional treatment and its impact on different treatment modalities, with the aim of providing new ideas and strategies for the clinical management of CD.

## CONCLUSION

Our results showed that additional VitD supplementation was safe and effective. The administration of VitD can not only relieve the inflammation state but also improve the immune environment of patients. Vitamin D promoted the restoration of Th17/Treg cell balance and lowered the level of CRP. This study confirmed the significant benefits of nutrition-related therapies in the prevention and treatment of disease, and additional administrated VitD can better improve clinical outcomes in patients with CD, undoubtedly providing a cost-effective alternative to clinical treatment.

## Figures and Tables

**Table 1.** Clinical Characteristics of Patients Between 2 Different Treatment Groups

Variable	Conventional Treatment	Vitamin D Treatment	*P*
Number of patients	52	50	
Age (years, mean ± SD)	28.1 ± 9.8	29.8 ± 10.7	.40
Gender (n, male/female)	27/25	28/22	.70
CDAI (mean ± SD)	311.9 ± 68.4	319.7 ± 72.7	.58
Duration of disease (n)			
<12 months	13	13	>.05
12-36 months	17	16	>.05
>36 months	22	21	>.05
Disease location (n)			
Terminal ileum (L1)	15	15	>.05
Colon (L2)	17	16	>.05
Ileal colon (L3)	20	19	>.05
Disease behavior (n)			
Non-stenosing, non-fistulizing (B1)	13	12	>.05
Stenosing (B2)	21	20	>.05
Fistulizing (B3)	18	18	>.05
Disease activity stage (n)			
Mildly active stage	13	12	>.05
Moderately active stage	23	22	>.05
Severely active stage	16	16	>.05
With perianal lesions (n)	19	17	>.05
Prior intestinal surgery (n)	6	7	>.05
Extraintestinal manifestations (n)	5	5	>.05

CDAI, Crohn’s disease activity index.

**Supplementary Table 1.** Laboratory Indexes and Body Mass Index Treated with VitD

Group	Time	ESR (mm/h)	CRP (mg/l)	BMI (kg/m^2^)	ALB (g/l)
RT group	before	38.98 ± 3.99	39.65 ± 7.02	18.01 ± 1.76	32.98 ± 1.77
	after	12.21 ± 1.87	18.91 ± 2.66	19.89 ± 2.51	36.87 ± 2.56
VitD group	before	36.43 ± 3.78	38.99 ± 7.23	17.92 ± 1.81	32.11 ± 1.62
	after	11.18 ± 1.62^*^	6.08 ± 2.72*#	20.53 ± 2.72*	36.72 ± 2.69*

*P <.05 versus before VitD treatment group; ^#^P <.05 versus after RT group.

RT, routine treatment; VitD, vitamin D; BMI, body mass index; ALB, albumin; ESR, erythrocyte sedimentation rate; CRP, C-reactive protein.

**Supplementary Table 2.** The Proportion of Th17 and Treg Cells Treated with VitD

Group	Time	Th17 (%)	Treg (%)	Th17/Treg
RT group	before	3.43 ± 0.98	3.88 ± 0.65	0.85 ± 0.21
	after	2.51 ± 0.89	4.55 ± 1.98	0.55 ± 0.11
VitD group	before	3.12 ± 0.93	3.56 ± 0.61	0.82 ± 0.19
	after	1.48 ± 0.62*	5.71 ± 2.53*	0.26 ± 0.12*^#^

*P <.05 versus before VitD treatment group; ^#^P <.05 versus after RT group.

RT, routine treatment; VitD, vitamin D; Th17, T helper cells; Treg, T regulatory cells.

**Supplementary Table 3.** The SES-CD, CDAI, IBDQ of Patients Treated with VitD

Group	Time	SES-CD	CDAI	IBDQ
RT group	before	7.8 ± 2.1	311.9 ± 68.4	138.9 ± 21.9
	after	4.8 ± 0.7	181.5 ± 47.8	161.6 ± 25.8
VitD group	before	7.9 ± 2.3	319.7 ± 72.7	137.8 ± 21.2
	after	3.9 ± 0.6*	179.6 ± 48.5*	158.1 ± 25.1*

*P <.05 versus before VitD treatment group.

RT, routine treatment; VitD, vitamin D; SES-CD, simple endoscopic score for Crohn’s disease; CDAI, Crohn’s disease activity index**;** IBDQ, Inflammatory Bowel Disease Questionnaire.
